# Dataset for adsorptive removal of tetracycline (TC) from aqueous solution via natural light weight expanded clay aggregate (LECA) and LECA coated with manganese oxide nanoparticles in the presence of H_2_O_2_

**DOI:** 10.1016/j.dib.2018.12.077

**Published:** 2018-12-28

**Authors:** Mohammad Noori Sepehr, Farideh Allani, Mansur Zarrabi, Mohammad Darvishmotevalli, Yasser Vasseghian, Saeid Fadaei, Mehran Mohammadian Fazli

**Affiliations:** a Department of Environmental Health Engineering, School of Public Health, Alborz University of Medical Sciences, Karaj, Iran; b Research Center for Health, Safety and Environment (RCHSE), Alborz University of Medical Sciences, Karaj, Iran; c Department of Health, Safety and Environment, Zanjan University of Medical Sciences, Zanjan, Iran; d Department of Environmental Health Engineering, Public Health School, Isfahan University of Medical Sciences, Isfahan, Iran; e Research Center for Environmental Determinants of Health (RCEDH), Kermanshah University of Medical Sciences, Kermanshah, Iran; f Department of Environmental Health Engineering, School of Public Health, Zanjan University of Medical Sciences, Zanjan, Iran

**Keywords:** Tetracycline, Light weight expanded clay aggregate, Adsorption, Manganese oxide, Aqueous solutions

## Abstract

In this data article, natural (NL) and manganese oxide-modified LECA (MML) adsorbents were applied for adsorptive removal of Tetracycline (TC) from aqueous solution. The used adsorbents was characterized using fourier transform infrared (FTIR) spectroscopy, scanning electron microscopy (SEM), X-ray diffraction (XRD) and X-ray fluorescence spectroscopy (XRF). The chemical analysis of XRF data revealed increased chemical composition of Mn as MnO to 8.96 wt%. The SEM patterns were illustrated the extent of surface and enhanced porosity in MML with Mn. In optimum operational conditions, maximum removal percentage of TC was achieved at 51.5 and 99.4% using NL and MML, respectively. The maximum adsorption capacities obtained from Langmuir modeling were 6.89 and 9.24 for NL and MML, respectively. The modeling of the adsorption kinetics revealed that TC adsorption by both NL and MML adsorbents was best-fitted with a pseudo-first-order model (*R*^2^ = 0.978). The isotherm studies of TC adsorption by MML showed that the Freundlich isotherm was the most appropriate model, with a higher coefficient of determination. The obtained data was illustrated that high competitive capacity of chloride and hardness ions compared with other ions against TC adsorption.

**Specifications table**

**Table:** 

Subject area	Environmental Health Engineering
More specific subject area	Environmental Chemistry
Type of data	Tables, figures
How data was acquired	In present data article, Tetracycline (TC) removal by natural (NL) and manganese oxide-modified LECA (MML) adsorbents was investigated in different rang of pH (3–11), adsorbent dosage (2–10 g L^−1^), initial TC concentration (10–50 mg L^−1^), contact time (1–200 min) and hydrogen peroxide (10–40 mL L^−1^) were studied on TC removal efficiency. Moreover, the obtained data were fitted by isotherms and kinetics equations.
Data format	Raw, analyzed
Experimental factors	All experiments were conducted in a batch reactor using classical approach.
Experimental features	Solutions preparation and sampling was done according to the standard method for water and wastewater treatment handbook [1]. In addition, TC concentration was measured using a Varian GBC Cintra10e UV–vis spectrophotometer at a maximum wavelength of 274 nm [2].
Data source location	Karaj city, Iran
Data accessibility	Data are included in this article
Related research article	Y. Gao, Y. Li, L. Zhang, H.Huang, J.Hu, S.M. Shah, X. Su, Adsorption and removal of tetracycline antibiotics from aqueous solution by graphene oxide, J. Colloid Interface Sci. 368(2012)540-6. (Published) [3].

**Value of the data**

• Due to cheap and high availability of this type of adsorbent in Iran, the efficiency of it can be improved by making these simple modifications and so the application of it in water and wastewater treatment will be increased.
• The obtained data of this study showed that manganese oxide modification effect on adsorbent led to increasing of equilibrium sorption capacity for removal of tetracycline.
• The obtained data of present dataset can be used for design and development of future similar studies. Because in this study, the optimal conditions for the removal of tetracycline by LECA and LECA coated with manganese oxide nanoparticles are determined. Therefore, the range of future study variables can be determined based on the optimal conditions of this dataset.

## 1 Data

**Fig. 1:**
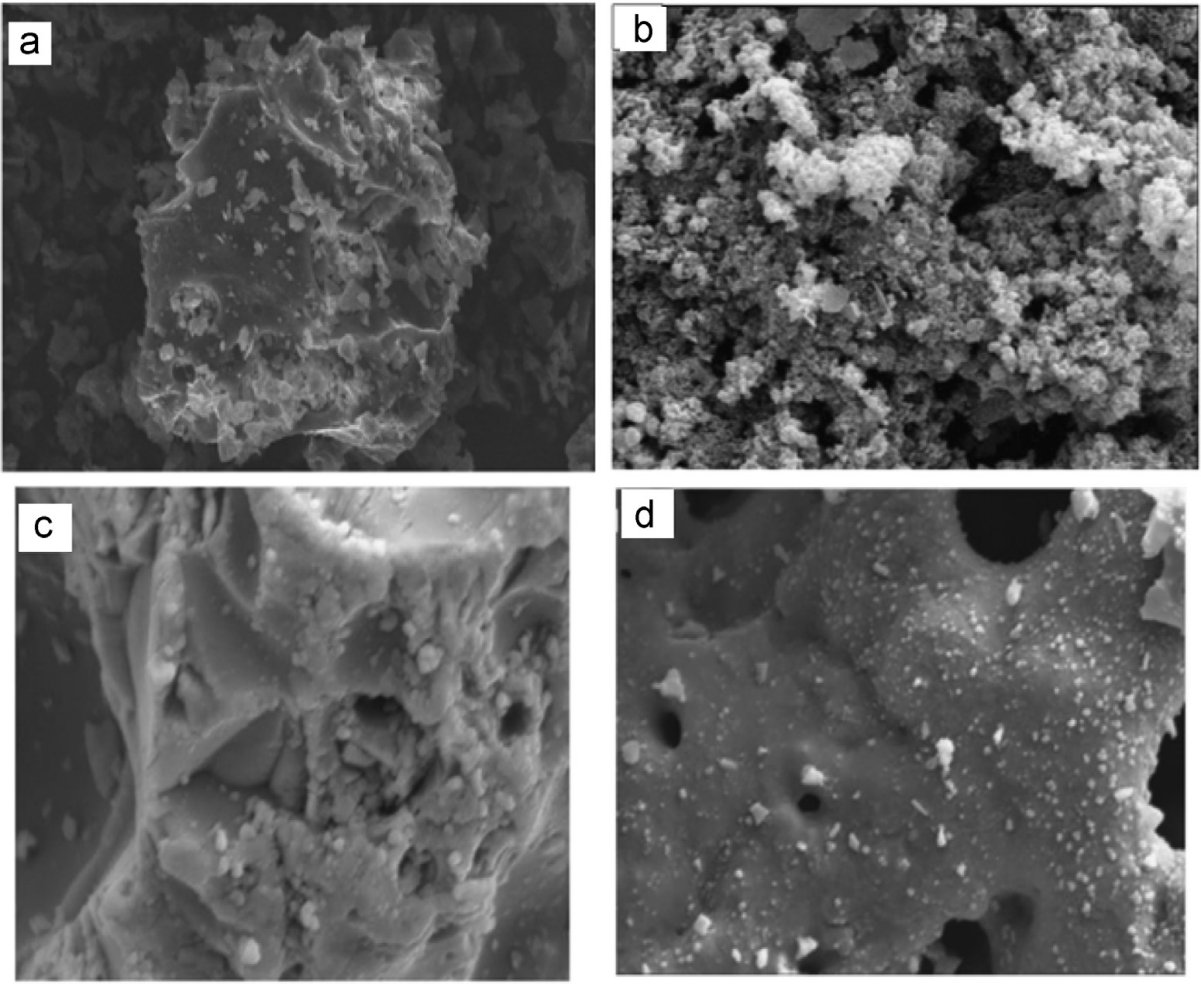
SEM images of (a) NL, (b) MML before and (c) NL, (d) MML after the adsorption of TC.

SEM images were used to observe the surface morphology of adsorbents. Fig. 1a–d shows the morphology of NL and MML, before and after TC adsorption and The XRF analysis of this adsorbent were shown in Table 1.

**Table 1:** Chemical compositions of natural LECA and manganese oxide-modified LECA (the 99.95% confidence limit).

**Component**	**Wt (%)**	
	**NL**	**MML**
SiO_2_	61.67	56.16
Al_2_O_3_	18.51	16.71
MgO	3.97	3.64
P_2_O_5_	0.19	0.21
SO_3_	0.23	0.18
K_2_O	3.28	2.96
CaO	3.50	3.17
TiO_2_	0.65	0*.5*8
Fe_2_O_3_	6.14	5.81
SrO	0.13	0.12
Na_2_O	1.54	1.45
MnO	–	8.96
SiO_2_/Al_2_O_3_	3.33	3.47

**Fig. 2:**
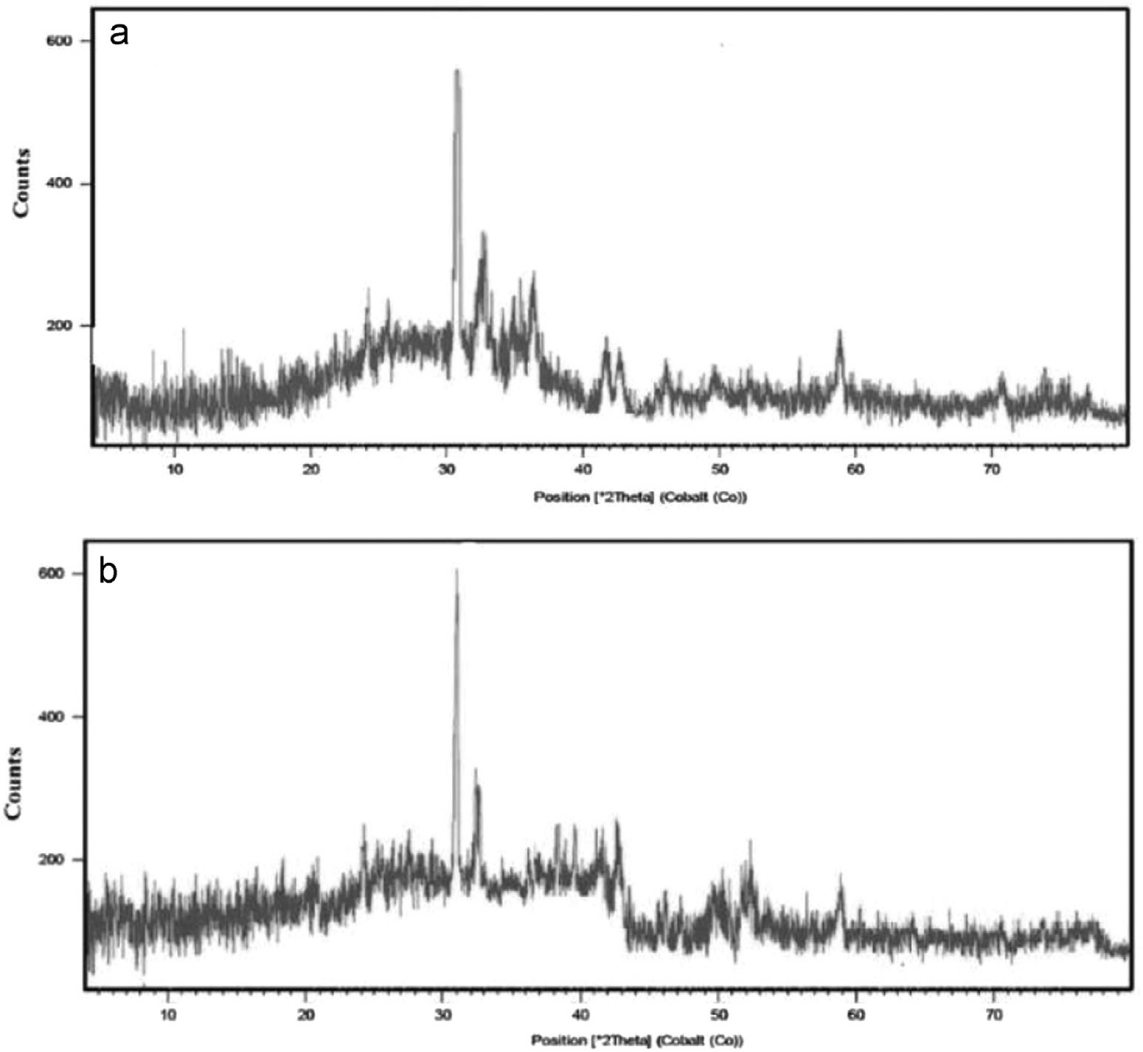
XRD patterns of (A) NL, (B) MML.

**Fig. 3:**
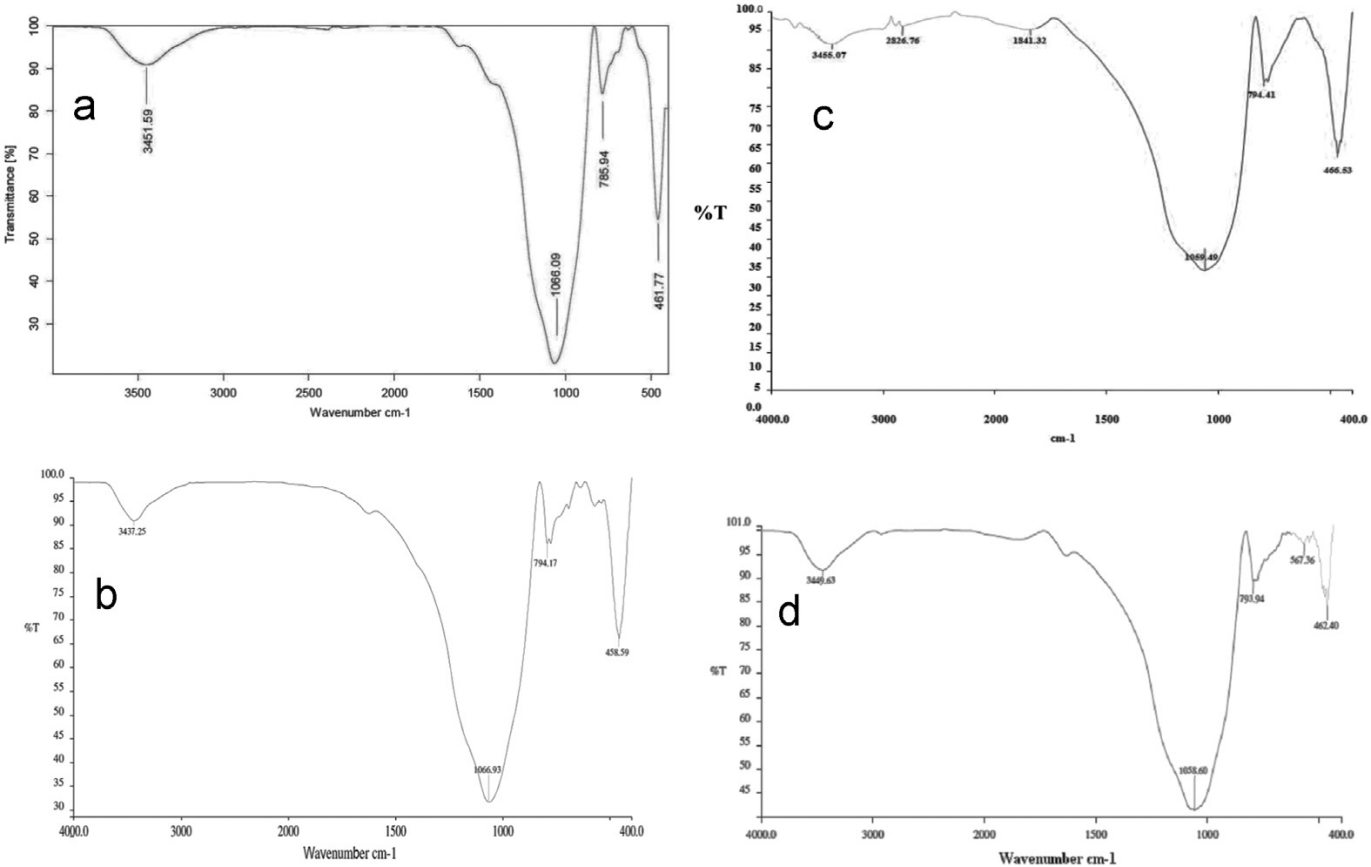
FTIR spectra of (a) NL, (b) ML before and (c) NL, (d) MML after the adsorption of TC.

**Fig. 4:**
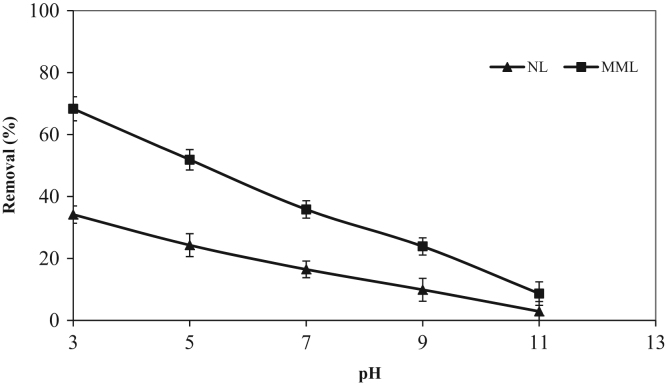
Effect of pH on TC removal by NL and MML. (TC concentration: 50 mg L^−1^; mass adsorbent, 2 g L^−1^; stirring rate, 250 rpm; temperature, 20 °C).

**Fig. 5:**
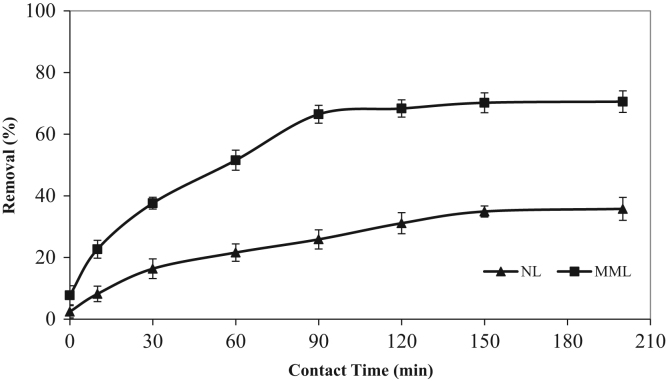
Effect of contact time on TC removal by NL and MML. (TC concentration: 50 mg L^−1^; mass adsorbent, 2 g L^−1^; stirring rate, 250 rpm; temperature, 20 °C, pH=3.0).

**Fig. 6:**
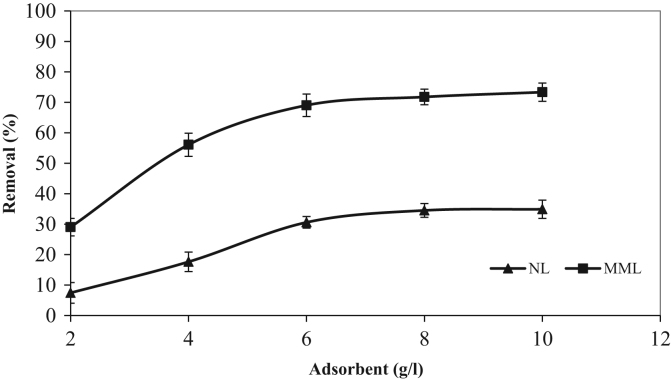
Effect of mass adsorbent on TC removal in the optimum operation conditions (conditions: pH, 3.0; contact time, optimum time for each adsorbent; stirring rate, 250 rpm; temperature, 20 °C).

**Fig. 7:**
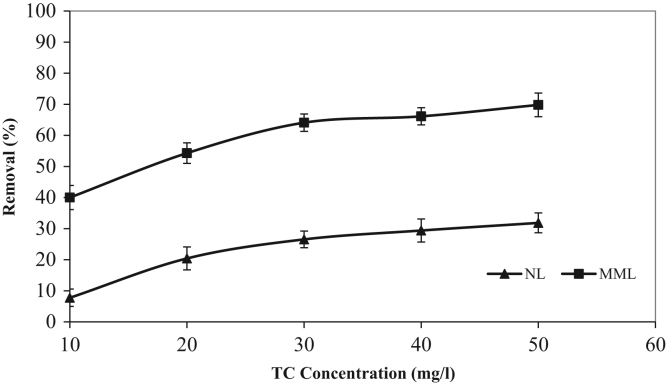
Effect of initial TC concentration on TC removal in the optimum operation conditions (conditions: pH, 3.0; contact time, optimum time for each adsorbent; stirring rate, 250 rpm; temperature, 20 °C).

**Fig. 8:**
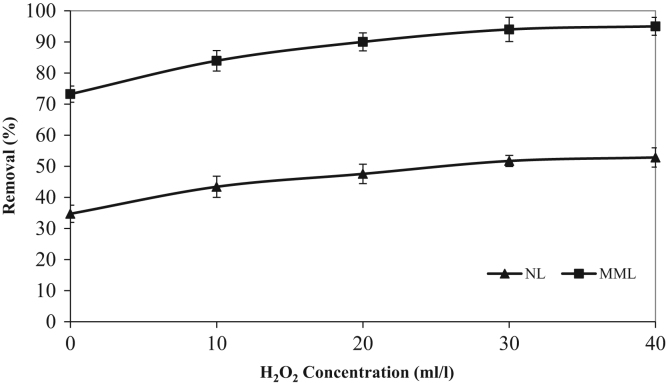
Effect of H_2_O_2_ concentration on TC removal in the optimum operation conditions (conditions: pH, 3.0; contact time, optimum time for each adsorbent; stirring rate, 250 rpm; temperature, 20 °C).

The XRD patterns of the NL and MML are displayed in Fig. 2a and b, confirming the mineralogical composition of adsorbents. A very broad peak and X high background are seen in Fig. 2, that indicated the presence of amorphous phase in the samples. Based on X-ray diffraction patterns data, majority of mineral phases that are involved in the adsorption process are minerals containing calcium and magnesium such as calcite (CaCO_3_), anorthite and dolomite. Anorthite is the calcium-rich member of the plagioclase solid solution series with the ideal formula of CaAl_2_Si_2_O_8_. The IR spectra of NL and MML, before and after TC adsorption, obtained in the range of 400–4000 cm^−1^ wavelength, are illustrated in Fig. 3a–d. In the present data article, the effect of solution pH on TC adsorption using two adsorbents is depicted in Fig. 4. It is recognized that the adsorption of TC by NL and MML is quite pH-dependent in the range of 3–11. It was observed that the adsorbed amount of TC under acidic condition was higher than alkaline condition for both adsorbents. Fig. 5 shows the influence of contact time on removal percentage of TC from aqueous solution. As shown in Fig. 5**,** TC uptake was increased with increasing contact time for both adsorbents and the highest sorption rate was observed for MML adsorbent. It was observed that the initial uptake rates were fast, as over 80% adsorption occurred within the first 90 min for both adsorbents. The obtained data was showed that for both adsorbents, the higher removal percentage was observed for 10 g L^−1^ adsorbent. As shown in Fig. 6, with increasing the adsorbent dosage from 2 to 8 g L^−1^, removal percentage of TC was increased from 7% to 35% and 30% to 69% for NL and MML, respectively. While, further increase in the adsorbent dosage above 8 g L^−1^ did not improved removal efficiency (Fig. 6). In order to find the influence of the initial TC concentration on the removal efficiency, several experiments were carried out at initial TC concentration, ranged from 10 to 50 mg L^−1^. As presented in Fig. 7, the removal efficiency was improved with an enhancement in the initial TC concentration (Fig. 7). As can be seen from Fig. 8, an increase in initial H_2_O_2_ concentration from 10 to 30 mL L^−1^ led to increasing removal efficiency of both adsorbents, as removal percentage of TC promoted from 43.6 to 51.5% and 88 to 99.4% for NL and MML, respectively.

**Fig. 9:**
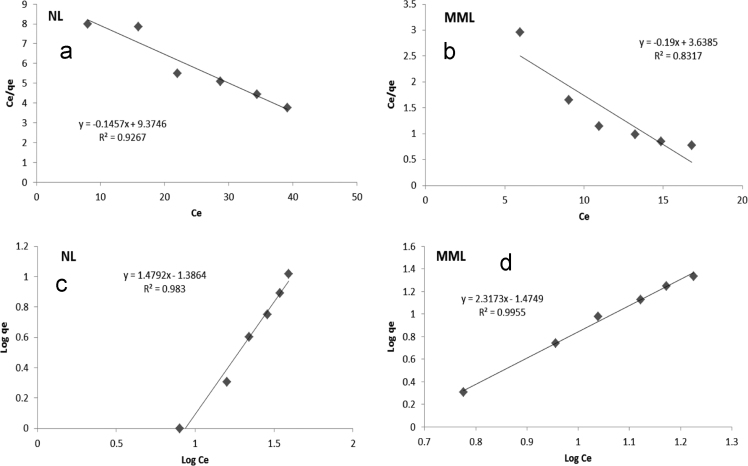
Langmuir (a) NL and (b) MML; Freundlich (c) NL and (d) MML adsorption isotherms for the adsorption of TC onto the adsorbent.

**Fig. 10:**
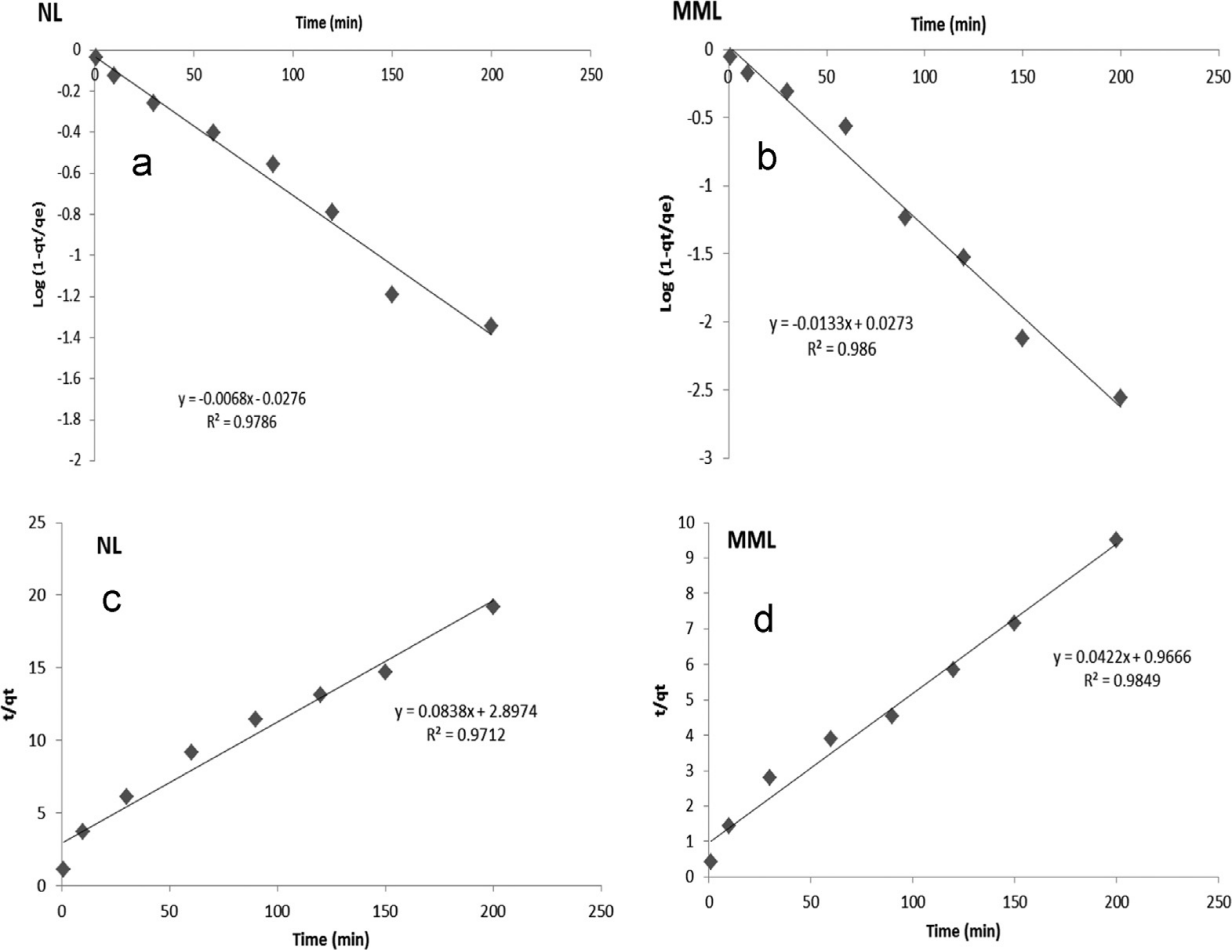
Kinetic data of TC adsorption onto adsorbents: pseudo-first-order model (a) NL and (b) MML; pseudo-second-order model (c) NL and (d) MML.

Table 2 shows isotherm parameters for NL and MML adsorbents and adsorption isotherms for the adsorption of TC onto the adsorbent were shown in Fig. 9. The modeling of the adsorption isotherms revealed that TC adsorption by both MML and NL adsorbents, with higher *R*^2^, was better fitted by Freundlich. The kinetic data of TC adsorption onto adsorbents was shown in Fig. 10 and the basic parameters were shown in Table 3. The modeling of the adsorption kinetics revealed that TC adsorption by both MML and NL adsorbents, with higher *R*^2^, was better fitted by pseudo-first-order model.

**Table 2:** Isotherm parameters for TC adsorption onto the NL and MML (the 99.90% confidence limit).

**Langmuir isotherm**						**Freundlich isotherm**					
**NL**			**MML**			**NL**			**MML**		
*q_m_* (mg g^−1^)	*b* (L mg^−1^)	R^2^	*q_m_* (mg g^−1^)	*b* (L mg^−1^)	*R*^2^	*K_F_* (mg/l)^−n^	1/n	*R*^2^	*K_F_* (mg/l)^−n^	1/*n*	*R*^2^
6.89	0.015	0.926	9.24	0.052	0.831	0.03	0.67	0.983	0.03	0.43	0.995

**Table 3:** Kinetic parameters for TC adsorption on NL and MML (the 99.95% confidence limit).

**NL**				**MML**			
**Pseudo-first- order**		**Pseudo- second- order**		**Pseudo-first- order**		**Pseudo- second- order**	
*k*_1_ (min^−1^)	*R*^2^	*k*_2_ (g mg^−1^ min^−1^)	*R*^2^	*k*_1_ (min^−1^)	*R*^2^	*k*_2_ (g mg^−1^ min^−1^)	*R*^2^
0.014	0.978	0.002	0.971	0.03	0.986	0.002	0.984

## 2 Experimental design, materials and methods

### 2.1 Synthesis of MML

The natural LECA (NL) used in present study, was purchased from Teb Azma Co. (Karaj, Iran). Natural LECA (NL) and manganese oxide-modified LECA (MML) were tested for TC adsorption from synthetic wastewater. First, to remove impurities from absorbent, it was washed with deionized water for several times until the turbidity value reached to less than 1 NTU. To evaporate the remaining water molecules, it was dried at 120 °C for 24 h. Then, adsorbent was milled and sieved to 10–30 meshes (841–2000 µm). Samples were kept in polyethylene containers until used directly to adsorb pollutants for coating by manganese oxide nanoparticles. The co-precipitation method was applied for synthesis of MML. The details of this method are well described in previous studies [4]. The experiments were performed in 500 mL sealed containers, so that 10 g NL was added to 200 mL deionized water. Then, 0.75 g cetyltrimethyl ammonium bromide and 3.2 g benzyl alcohol were added to this mixture. To obtain a homogeneous solution, mixture was stirred for 30 min. Then, 3.2 g potassium permanganate was added to solution and stirred until the formation of a brown precipitate. To further complete the reaction, solution was shaken for 24 h. After that, brown precipitate of MML was separated from mixture and washed several times with deionized water to remove uncoated MnO_2_ particles. Finally, calcination was carried out at 350 °C for 24 h to stabilize MML. Samples were kept in special containers to be used for the adsorption experiments.

### 2.2 Adsorbate preparation

All chemicals used in present study were of analytical reagent grade and used without further purification. TC stock solution was prepared by dissolving 0.121 g TC (obtained from Genview Chemical Co.) in solution containing 10 mL methanol, and then the volume of solution reached to 2000 mL by deionized water. Stock solution was prepared with containing 60 mg L^−1^ TC that was stored in a glass container at room temperature. Desired concentrations of TC were obtained by diluting the stock solution. Because of the instability of TC solution, the working solutions were instantaneously prepared for each experimental series. The characteristics and chemical structure of TC are shown in Table 4 [2].

**Table 4:** Characteristics and chemical structure of TC.

### 2.3 Characterizations of prepared adsorbent

In this study, the surface morphology of adsorbents was observed using scanning electron microscope (SEM) analysis in a LEO 1450 VP (England) operated at an acceleration voltage of 20 kV, with Au sputtering-coated samples fixed in an Al stub. The FT-IR spectrum was recorded (Model: Bruker-VERTEX 70, Germany) as a technique in order to know the nature of the active groups responsible for the adsorption process. The infrared (IR) spectra of the samples were obtained within the wave number range of 400–4000 cm^−1^. The chemical composition of the adsorbents was determined by means of an X-ray fluorescence spectroscopy (XRF) instrument (Philips-Magix Pro., Philips Electronics Co., Netherlands). The X-ray diffraction (XRD) patterns of two adsorbents were collected by means of a PHILIPS Xpert pro equipped with a Cu Kα as a radiation source (1.54056 Å) generated at 40 kV and 40 mA.

### 2.4 Batch adsorption experiments

All experiments were conducted in a batch reactor using classical approach. The effects of experimental parameters including, pH (3–11), adsorbent mass (2–10 g L^−1^), initial TC concentration (10–50 mg L^−1^), contact time (1–200 min) and hydrogen peroxide (10–40 mL L^−1^) were studied on TC removal efficiency. The experiments were carried out in 250 mL flasks containing 100 mL of TC solution at room temperature. To study the effect of pH on the removal of TC, pH values were changed from 3 to 11 at flasks containing adsorbent dosage of 2 g L^−1^ and 50 mg L^−1^ of TC solution. For this purpose, flasks were shaken at 250 rpm for a predetermined time period. The influence of time on the removal efficiency was investigated by mixing 2 g L^−1^ adsorbent dosage with TC concentration of 50 mg L^−1^ at a fixed pH 3.0. Flasks were placed on a shaker with constant speed of 250 rpm. The samples were taken at a predetermined time interval and analyzed for their TC concentration. The results obtained from this stage were used for kinetic studies. To investigate the effect of varying adsorbent dosages on TC removal efficiency, 100 mL of TC solution (50 mg L^−1^) was added to each flasks containing different values of adsorbent. In this stage, pH and contact time were optimized from previous tests. In addition, the adsorption equilibrium studies were conducted at pH 3.0 with initial concentration of TC ranging from 10 to 50 mg L^−1^ and the adsorbent mass 2 g ^−1^ L. The influence of different concentrations of hydrogen peroxide was evaluated in the optimum conditions obtained from previous steps. In order to perform this experiment, flasks containing different concentrations of hydrogen peroxide were placed on a shaker with constant speed of 250 rpm. The effects of competing ions including chloride, nitrate, and sulfate and hardness on the removal efficiency, were examined by using 250 mL flasks containing 100 mL of desired competitor ion (at predefined concentration) and 50 mg L^−1^ TC. The mixture was placed in the shaker with constant speed of 250 rpm for a predetermined time interval. For all experiments, samples were taken at desired time intervals and the adsorbent was separated from the aqueous solution by filtration (Whatman, 0.45 μm) and centrifuged (Sigma-301, Germany). Residual TC concentration was measured using a Varian GBC Cintra10e UV–vis spectrophotometer at a maximum wavelength of 274 nm.

### 2.5 Adsorption isotherms and kinetics

The adsorbent capacity could be described using sorption isotherm. In the present study the adsorption data of TC were evaluated by Langmuir and Freundlich isotherms. The linear Langmuir isotherm presented as fallow [5]:

(1) $$\frac{C_{e}}{q_{e}}=\;\frac{1}{bq_{m}}+\;\frac{C_{e}}{q_{m}}$$

where the *C_e_* is equilibrium concentration (mg/l), *q_e_* is TC adsorbed at equilibrium (mg/g), *q_m_* and *b* are the Langmuir constants related to the capacity and energy of adsorption, respectively [6], [7], [8].

Freundlich adsorption isotherm is an empirical expression that describes adsorption on a heterogeneous surface. The linear Freundlich isotherm could be illustrated as fallow:

(2) $$\ln q_{e}=\ln k_{f}\;+\;n^{-1}{\mathrm{lnC}}_{e}$$

where *K_f_* and *n* are Freundlich constants corresponded to adsorption capacity and adsorption intensity, respectively [9], [10], [11]. The kinetics was investigated via adsorption of certain concentration of TC at different contact time. Kinetic study is essential for providing information on the factors affecting it reaction speed.

Several kinetics includes pseudo-first-order, pseudo-second-order, intraparticle diffusion of Morris and Weber and elovich (the elovich equation is suitable for systems with heterogeneous adsorbing surfaces) [12] were used to controlling mechanisms of the adsorption process. The equations of kinetic models are expressed as follows [13], [14], [15], [16]:

Pseudo-first-order:

(3) $$\ln\left(q_{e}-q_{t}\right)=\ln q_{e}-k_{1}t$$

Pseudo-second-order:

(4) $$\frac{1}{q_{t}}=\frac{1}{q_{e}}\;+\;k_{2}t$$
