# Supplementary material for: Dataset for adsorptive removal of tetracycline (TC) from aqueous solution via natural light weight expanded clay aggregate (LECA) and LECA coated with manganese oxide nanoparticles in the presence of H2O2
Source: Data Brief. 2018 Dec 28;22:676–86. doi: 10.1016/j.dib.2018.12.077 (PMC6327738; doi:10.1016/j.dib.2018.12.077)
Supplement: Supplementary file 1 — Transparency document [file mmc1.docx]

Dear editor(s)

Data in Brief

Greetings!

At this time, this article with entitled ***“Dataset for adsorptive removal of tetracycline (TC) from aqueous solution via natural light weight expanded clay aggregate (LECA) and LECA coated with manganese oxide nanoparticles in the presence of H2O2 “*** has not been published or submitted in any other journals. We hope that the results of this research will be of interest to you. None of the authors of this paper have any conflicts of interest to declare.

**Best regards**

***Corresponding author:** Mehran Mohammadian Fazli
